# Spatial and Seasonal Dynamic of Abundance and Distribution of Guanaco and Livestock: Insights from Using Density Surface and Null Models

**DOI:** 10.1371/journal.pone.0085960

**Published:** 2014-01-22

**Authors:** Natalia M. Schroeder, Silvia D. Matteucci, Pablo G. Moreno, Pablo Gregorio, Ramiro Ovejero, Paula Taraborelli, Pablo D. Carmanchahi

**Affiliations:** 1 CONICET, Instituto Argentino de Investigaciones de las Zonas Áridas (IADIZA), Mendoza, Argentina; 2 CONICET, GIEFAS-INIBIOMA-AUSMA-UNCo, San Martín de los Andes, Neuquén, Argentina; 3 CONICET, GEPAMA-UBA, Buenos Aires, Argentina; 4 CONICET, ICiVet-Litoral, LEcEn, FCV-UNL, Santa Fe, Argentina; 5 CONICET, GIB-IADIZA, Mendoza, Argentina; Université de Sherbrooke, Canada

## Abstract

Monitoring species abundance and distribution is a prerequisite when assessing species status and population viability, a difficult task to achieve for large herbivores at ecologically meaningful scales. Co-occurrence patterns can be used to infer mechanisms of community organization (such as biotic interactions), although it has been traditionally applied to binary presence/absence data. Here, we combine density surface and null models of abundance data as a novel approach to analyze the spatial and seasonal dynamics of abundance and distribution of guanacos (*Lama guanicoe*) and domestic herbivores in northern Patagonia, in order to visually and analytically compare the dispersion and co-occurrence pattern of ungulates. We found a marked seasonal pattern in abundance and spatial distribution of *L. guanicoe*. The guanaco population reached its maximum annual size and spatial dispersion in spring-summer, decreasing up to 6.5 times in size and occupying few sites of the study area in fall-winter. These results are evidence of the seasonal migration process of guanaco populations, an increasingly rare event for terrestrial mammals worldwide. The maximum number of guanacos estimated for spring (25951) is higher than the total population size (10000) 20 years ago, probably due to both counting methodology and population growth. Livestock were mostly distributed near human settlements, as expected by the sedentary management practiced by local people. Herbivore distribution was non-random; i.e., guanaco and livestock abundances co-varied negatively in all seasons, more than expected by chance. Segregation degree of guanaco and small-livestock (goats and sheep) was comparatively stronger than that of guanaco and large-livestock, suggesting a competition mechanism between ecologically similar herbivores, although various environmental factors could also contribute to habitat segregation. The new and compelling combination of methods used here is highly useful for researchers who conduct counts of animals to simultaneously estimate population sizes, distributions, assess temporal trends and characterize multi-species spatial interactions.

## Introduction

Large native herbivores are relatively difficult to conserve because of their wide home ranges and tendency to come into conflict with humans, since they share similar dietary preferences with domestic herbivores [1], [2]. The greatest challenge in managing and conserving large herbivores is monitoring their populations, which is crucial when assessing both the success of management actions and formulating new strategies. The latter is particularly relevant in the case of the guanaco (*Lama guanicoe*), which has recently been proposed as a “pest species” by the Santa Cruz province legislature (southern Argentina), which is requesting mitigation actions for population control although having no reliable estimates of abundance. This initiative has led to strong rejection by specialists and national and international institutions (Appendix S1).

Until recently, obtaining un-biased estimates of the spatial and seasonal dynamic of abundance and distribution of large herbivores at ecologically meaningful scales required a huge sampling effort. The advancement of statistical tools combined with geographic information systems has led to new methods for analyzing, modeling and predicting species distribution and abundances at larger scales. Following a model-based approach, density surface models (DSM) allow the modeling of spatial variation in density using the traditional line transect methodology, but combining the fundamentals of distance sampling based on the probability of detection [3] with generalized additive models (GAM) [4]. Unlike the traditionally design-based approach, the major advantage of DSM is that it allows the estimation of abundance in any sub-area of interest and also assess spatial distributions of that abundance [3]. This has very important implications for conservation and management of a wide range of species. For example, DSM can be used to identify specific locations where human impact on vulnerable species should be minimized, even if no overall change in abundance occurs [5], or to identify a differential spatial distribution of age classes associated with different types of habitats [6]. DSM has been successfully applied to know spatial distribution abundance of aquatic molluscs [6], [7], marine mammals [8] and seabirds [9], but to our knowledge, no studies have proven this methodology for terrestrial fauna.

Monitoring herbivore population dynamics is also useful in order to better understand diverse ecological processes at landscape and ecosystem levels [10], [11]. Co-occurrence patterns among species can be used to infer mechanisms of community organization, such as biotic interactions. For example, under the hypothesis of interspecific competition, species are expected to co-occur less often than expected by chance (segregation pattern) with the opposite being expected for facilitative interactions (aggregation pattern). Null models have been a popular tool for detecting species co-occurrence patterns, typically from data in the form of binary presence/absence matrices [12]. However, if species abundances are known, more powerful null models of community assembly can be built [13]. Moreover, the combination of null modeling with count data from a distance sampling surface approach has not yet been used in field research.

*Lama guanicoe* was the dominant herbivore of arid lands in southern South America, but has suffered a drastic decline in its abundance and distribution range since the 1800s [14]. Although it is not considered endangered at a continental scale, *L. guanicoe* is currently ecologically extinct in most of its remaining range [15]. Much of the species decline has been the result of over hunting and introduction of livestock, mainly sheep, as an extensive productive activity [14], [16]. La Payunia Reserve in northern Patagonia of Argentina is the protected area supporting the highest number of guanacos across their current distribution range and the least disturbed populations in Patagonia [17], [18]. Since 1982, the guanaco population has been periodically monitored, observing a recovery in its density [18], although there have been no empirical abundance estimates in the past 20 years. At the same time, despite the fact that competition for food with livestock has been suggested as one of the major threats to guanaco population, knowledge of livestock abundance and distribution is also scarce and fragmented. Much of this lack of information is because official statistics on livestock numbers and distribution are not available to the public. The guanaco distribution in relation to other domestic herbivores has been traditionally focused on sheep [16], [19], [20], which share more similarities in diet and feeding styles with the native species [17]. The interaction of guanaco with other domestic herbivores with different feeding styles and larger body sizes, such as horses and cattle, has received little attention, or has been based on presence-absence data [21]. For a social species like *L. guanicoe*, which lives in different kind of groups all year round [22], co-ocurrence patterns with other species could be more accurate and better understood through the use of abundance, instead of binary presence-absence data.

In this study, we combine density surface and null models of abundance data as a novel approach to analyze the spatial and seasonal dynamics of abundance and distribution of guanacos and domestic herbivores in northern Patagonia, in order to visually and analytically compare the dispersion and co-occurrence pattern of ungulates. The objectives were to (a) update the information on total abundance of guanacos in the study area, 20 years after the last estimates, (b) analyze the seasonal patterns of the abundances, (c) provide systematized information on livestock numbers and spatial and seasonal distribution for the first time, and (d) analyze spatial and seasonal co-occurrence patterns of herbivores. *L.guanicoe* overlaps its diet by 68% with horses, 56% with cattle, 48% with goats and 82% with sheep. In turn, guanacos (100–120 kg), goats (50 kg) and sheep (50 kg) are classified as small grazer-browser ungulates, while horses (300 kg) and cattle (350 kg) are large strictly-grazer ungulates [17]. Therefore, we hypothesized that ecologically similar herbivores, in terms of body weight, diet and feeding styles, would show spatial segregation patterns to meet nutritional requirements. Consequently, we expect to find (1) high spatial segregation between guanacos and domestic herbivores in general and (2) higher spatial segregation between guanacos and small-livestock (goats and sheep) than between native species and large-livestock (cattle and horses).

## Methods

### Ethics statements

The present work is a non-invasive study, conducted through the observation of animal groups using binoculars. Permission for the research was given by the Direction of Natural Renewable Resources of Mendoza Province (DRNR, Resolutions n° 117/09 and 795/10).

### Study area and field work

**Figure 1:**
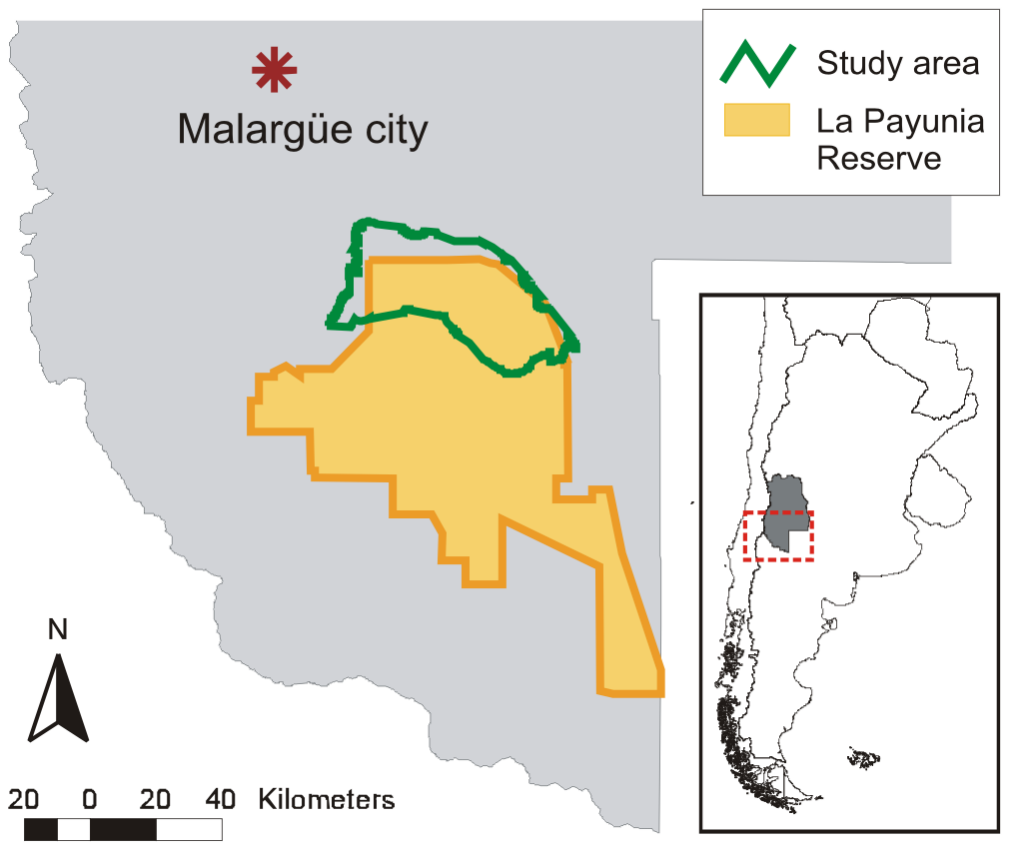
Study area in the northern part of La Payunia Reserve and surrounding area. It is located in Mendoza province, central-west Argentina.

This study was conducted in northern Patagonia, central-west Argentina, during 2 austral springs (October 2008, December 2009), 2 summers (January 2009 and February 2010), 2 falls (May 2009, April 2010) and 3 winters (June and September 2009, July 2010). The study area of 1671 km^2^ is located between 36°00' and 36°36' S, and 68°34' and 69°23' W, including the northern part of the 6641 km^2^ La Payunia Reserve (Fig. 1). It is dominated by a gently undulating relief and vast flatlands, combined with steeper hills and volcanic outcrops. Temperatures average 6°C (winter) and 20°C (summer), and annual precipitation is scarce (198 mm). The vegetation is xerophytic, with low cover (58%), belonging to La Payunia phytogeographic province within the Andean–Patagonian domain [23]. Sandy plains are covered by herbaceous communities dominated by *Panicum urvilleanum*, *Stipa speciosa* and *Sporobolus rigens*, while slopes and basaltic scoria present shrub communities mainly of *Neosparton aphyllum* and *Ephedra ochreata.*

### Animal surveys

Ground surveys of herbivores were made traveling on accessible roads and tracks, totaling the same 180 km of transects per visit, 10 km on average and spaced by 1 km. We followed the line transect method [24], which consists of observations made from a pick-up vehicle driven at an average speed of 30 km/h. We recorded the number of guanacos, type and number of livestock (goats, cattle, sheep and horses), distance and angle from the observer to the group, measured with a laser rangefinder and compass, respectively. We estimated the perpendicular distance for each observation to the transect line with the angle and distance from the observer to the group for subsequent analyzes. Summary of animal observations recorded in the line transects is detailed in Table 1.

**Table 1:** Summary of the number of groups and total individuals for *L. guanicoe* and domestic ungulates in the 9 surveys.

		Number of groups (total individuals)^a^				
Season	Date	lg	h	c	gt	s
Spring	October 2008	290 (4051)	22 (101)	74 (540)	13 (939)	2 (22)
	December 2009	259 (3364)	11 (65)	48 (453)	8 (428)	-
Summer	January 2009	554 (2625)	19 (90)	67 (388)	-	-
	February 2010	592 (4238)	23 (115)	85 (561)	7 (222)	3 (77)
Fall	May 2009	156 (1191)	39 (162)	72 (281)	6 (230)	3 (62)
	April 2010	97 (1656)	35 (166)	57 (386)	5 (268)	-
Winter	June 2009	78 (2128)	30 (150)	55 (319)	4 (281)	2 (8)
	September 2009	175 (3218)	24 (180)	54 (318)	5 (241)	5 (112)
	July 2010	56 (911)	31 (147)	28 (216)	2 (124)	3 (62)
	**TOTAL**	**2257 (23382)**	**234 (1176)**	**540 (3462)**	**50 (2733)**	**18 (343)**

a After removing 5% of extreme distance values in raw data (see text for details).

References: lg: guanaco; h: horses; c: cattle; gt: goats; s: sheep.

### Detection function and density surface model (DSM)

The detection function, *g(y)*, estimates the probability of detecting an animal at a distance *y* from the transect line. It was calculated from perpendicular distances of each field observation. Raw data were truncated to remove 5% of extreme distance values to reduce detectability error [24]; resulting truncation distance was *w  = * 800 m (guanaco and large-livestock) and *w  = * 900 m (small-livestock). A Hazard-rate key with two parameters was used to adjust a detection function (Appendix S2, Table S1 and Fig. S1).

We grouped cattle and horses as large-livestock, and goats and sheep as small-livestock following Puig and collaborators [17] in order to increase sampling size. We divided each transect into smaller segments of 2 km in length (*l*), which is approximately equal to twice the truncation distance *w* for all herbivores [3]. We recorded the number of animals within each segment as *n_i_, i  =  1,...T,* totaling 89 segments for the whole surveyed area.

We used three spatial covariates to model the density surface of ungulates in the study area: geographic coordinates (latitude (*y*), longitude (*x*)) and distance from human settlements (*d*) of each segment, all expressed in meters. Location data of human settlements was obtained from own records and information from park rangers.

We did not find multicollinearity among predictor variables (Pearson’s correlation value, |r|, was <0.6 for all combinations).

The total number of animals within a segment *i* was estimated using a Horvitz-Thompson-like estimator [3]:
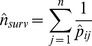



where 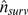 is the number of animals in the surveyed area, *n* is the number of animals seen, and 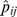 is the estimated probability of observing an animal *j* in a segment *i*, obtained from the detection function.

We used generalized additive models (GAMs) [25], with quasi-poisson error distribution and logarithmic link function to relate the estimated abundance values in each segment (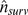) with spatial covariates. The optimal degrees of freedom (*gl*) for the smoothed function in GAMs were defined by generalized cross validation (GCV), but we fixed the maximum possible value at *gl*  =  5 (knots *k = *6) for geographic variables (*x, y*), and at *gl = *3 (*k = *4) for variable *d*, to prevent model overfitting [25]. We selected the best-fit model based on the lowest GCV value.

To assess temporal trends in abundance and spatial density distribution, we selected 9 DSMs for guanaco and large-livestock, one per survey. For small-livestock, we only fitted an annual DSM due to small sample size.

### Abundance estimation and spatial density distribution maps

Estimating densities by using a model-based approach as DSM represents a variation in the traditional method for sampling distances, which relies on sampling design (design based approach) in order to guarantee unbiased estimates [3]. In contrast, spatial modeling does not require that transects be located according to a formal sampling scheme (i.e. random or stratified). This is useful when the viable option is a nonrandom ground sampling on existing roads or tracks. However, this approach does not correct for the bias of following the geography of roads, which complicates the model’s predictive capacity toward sites outside such geography. Taking this limitation into account, we defined the prediction area as those sites meeting the following criteria: (a) roads and tracks in the northern zone of La Payunia Reserve and surrounding areas, obtained from National Geographic Institute database, supplemented with our own and rangers’ records; (b) 2 km on both sides of the roads and tracks, which is approximately the maximum distance at which detection is possible, totaling 4 km of strip width, and (c) areas between 1350 and 1800 m.a.s.l., which is the elevation interval encompassed by the surveyed transects, using a Digital Elevation Model obtained from the Global Land Cover Facility at the University of Maryland (http://glcf.umd.edu/). We obtained a prediction area of 1220 km^2^ (representing 73% of the 1671 km of total study area), which was then divided into a grid of 305 square cells of 4 km^2^ each.

We estimated the total abundance of ungulates in the prediction area as the sum:
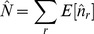



where 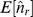 are animal numbers predicted by the selected DSM in each 4 km^2^ cell *r* of the prediction grid. We then created a density distribution map for each group of herbivores and survey.

The variance in the estimation of abundance has two components. The first one, arising from the detection function, was obtained according to Buckland and collaborators [26], whereas the second component derived from the DSM model was calculated using a moving window parametric resampling technique [27], with 99 replications and 2 segments as sampling unit (*m*). We assumed that *m* = 2 was large enough to ensure independence between segments which were two units apart.

GIS analyses were performed in ArcView 3.2; detection function and DSM were fitted in Distance 6.0 (http://www.ruwpa.st-and.ac.uk/distance/), which uses the R *mgcv* package [25] (www.r-project.org).

### Co-occurrence patterns from null model analysis

Abundance data resulting from the prediction grid were then organized as abundance matrices, in which rows represent species and columns represent the 305 grid cells. We compared co-ocurrence patterns separately for *L.guanicoe* and large-livestock, and for *L.guanicoe* and small-livestock, for each survey. For small-livestock, we used the same annual abundance data for all matrices. A total of 16 matrices were analyzed. We calculated the *U-*ratio  =  *V*/*W* [13] as a metric of co-occurrence, which compares the variance of row totals *V* with the sum of the column variances *W*. Low values of *U* indicate negative covariation in abundance between species. If species are segregated, we expect the *U*-ratio to be smaller than expected by chance. We calculated the observed *U*-ratio for each abundance matrix and compared it with the expected *U*-ratio (*Ū*), calculated for 5000 randomly null matrices using the IT algorithm. This algorithm assigns individuals randomly to matrix cells with probabilities proportional to the observed row and column abundance totals until, for each row and column, total abundances are reached [13]. The combination of *U*-ratio and IT algorithm has proved to outweigh other combinations tested in co-occurrence analyzes with abundance matrices [13]. To asses our prediction that *L.guanicoe* is more spatially segregated from small-livestock than from large-livestock, we calculated the standardized effect size (SES) as (*U*–*Ū*)/sd*_Ū_*, were sd*_Ū_* is the standard deviation of 5000 simulated *U*-ratio (*Ū*). Here, the greater the SES, the higher the segregation pattern among species. All analyzes were executed in R package *stats* (www.r-project.org).

## Results

### Density surface models

The deviation explained by the best-fit DSMs for *L.guanicoe* varied from 18.1% in July 2010 to 50.4% in February of the same year (Table 2). Except for summer surveys, the best models included only pure spatial variables (latitude and longitude). For large-livestock, best-fit models explained a high percentage of the deviation, which exceeded 42% in all surveys (Table 2), although it was not possible to fit a DSM for October 2008 because none yielded reliable predictions of abundance. Best-fit annual DSM for small-livestock explained a deviation of 38.1% (Table 2). Except for summer 2010 for large-livestock (Table 2), the best-fit models for both types of livestock also included the distance to human settlements as a predictor variable. Details of all density surface models tested for herbivores are given in Table S2.

**Table 2:** Density surface models (DSM) selected for herbivores in all surveys.

	Survey	Model	Co-variables	Expl. Dev. (%)
*Lama guanicoe*	October2008 (spring)	hg1	s(*x*) +s(*y*)	28.2
	January 2009 (summer)	hg5	s(*x*) +s(*y*) + s(*d*)	43.7
	May 2009 (fall)	hg7	s(*x*) +s(*y*)	24.3
	June 2009 (winter)	hg10	s(*x*) +s(*y*)	50.2
	September 2009 (winter)	hg13	s(*x*) +s(*y*)	36.5
	December 2009 (spring)	hg16	s(*x*) +s(*y*)	27
	February 2010 (summer)	hg20	s(*x*) +s(*y*) + s(*d*)	50.4
	April 2010 (fall)	hg22	s(*x*) +s(*y*)	31.4
	July 2010 (winter)	hg25	s(*x*) +s(*y*)	18.1
*Large-livestock*	January 2009 (summer)	hm5	s(*x*) +s(*y*) + s(*d*)	51.6
	May 2009 (autumn)	hm8	s(*x*) +s(*y*) + s(*d*)	65.3
	June 2009 (winter)	hm11	s(*x*) +s(*y*) + s(*d*)	66.2
	September 2009 (winter)	hm14	s(*x*) +s(*y*) + s(*d*)	42.8
	December 2009 (spring)	hm17	s(*x*) +s(*y*) + s(*d*)	43.3
	February 2010 (summer)	hm19	s(*x*) +s(*y*)	47.8
	April 2010 (autumn)	hm23	s(*x*) +s(*y*) + s(*d*)	54.6
	July 2010 (winter)	hm26	s(*x*) +s(*y*) + s(*d*)	56
*Small-livestock*	Annual	he2	s(*x*) +s(*y*) + s(*d*)	38.1

Percentage of explained deviance are given.

### Estimated abundances and seasonal trends

**Figure 2:**
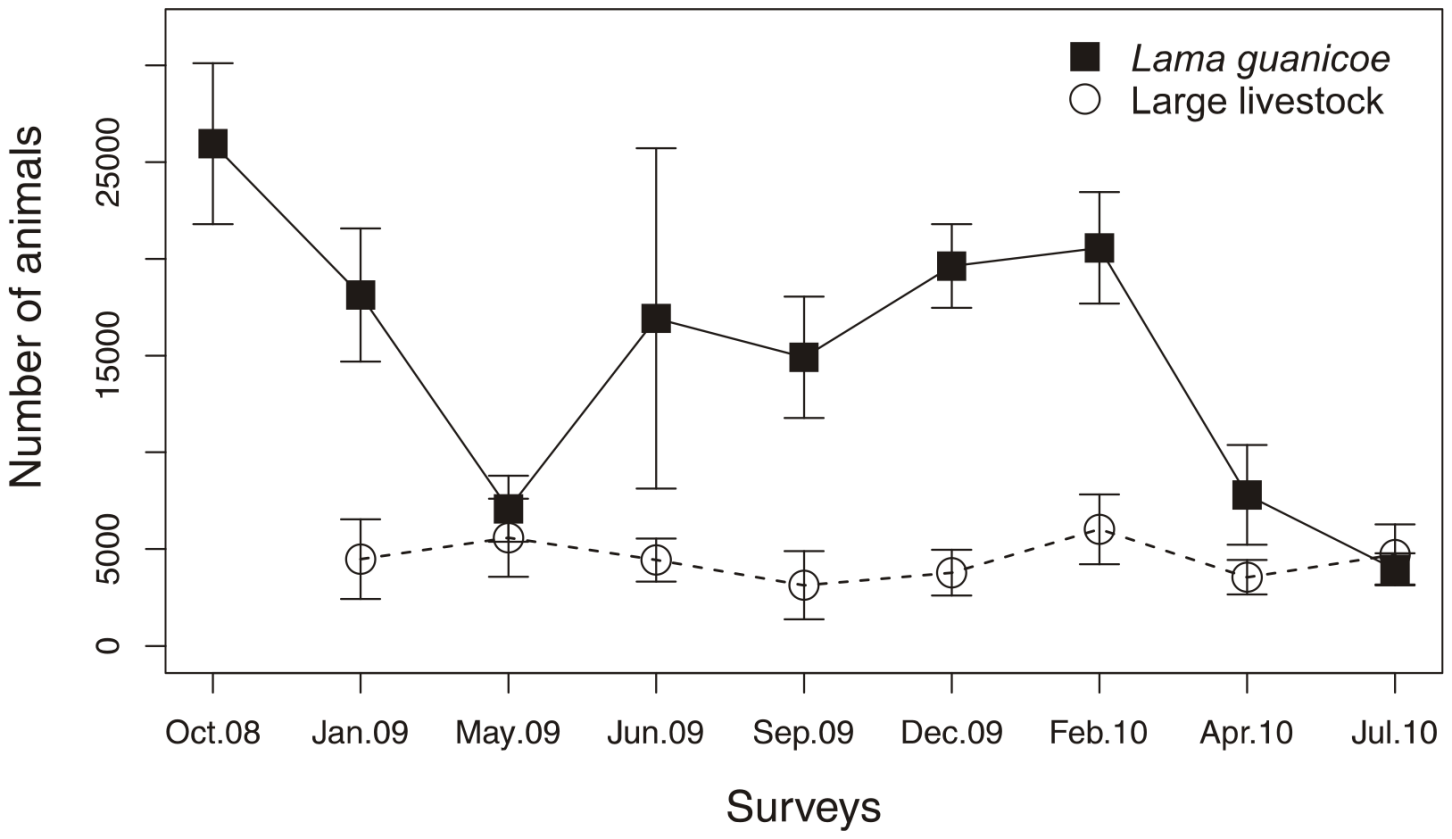
Seasonal abundance for *Lama guanicoe* and large-livestock during the 9 surveys. The bars correspond to standard deviation.

Guanaco abundance varied seasonally, from a minimum of 3961 (CV  =  21%) animals in July 2010 (winter) to a maximum of 25951 (CV  =  16%) animals in October 2008 (spring) (Table 3). Globally, guanacos were more abundant in spring and summer, and less abundant in winter and autumn, although an important inter-annual variability was observed (Fig. 2). In contrast, large-livestock abundance was more constant throughout the year (Fig. 2), with a minimum of 3139 (CV  =  56%) animals in September 2009 (winter) and a maximum of 6022 (CV  =  30%) in February 2010 (summer), although variation coefficients were high in some surveys (Table 3). The annual average of guanacos was 14995, similar to that of domestic herbivores (14026), of which more than half were goats and sheep (9611) (Table 3).

**Table 3:** Estimated abundances for *L. guanicoe* (g), large-livestock (la), and small-livestock (sm), according to the best-fit DSMs.

Season	Survey	Estimation of abundance (N)	Total CV	Confidence interval (95%)	Average density (indiv./km^2^)
Spring	October 2008	25951 (g)	0.16	19004; 35436	21.27
		-	-	-	-
	December 2009	19627 (g)	0.11	15689; 24555	16.09
		3786 (la)	0.31	2108; 6800	3.06
Summer	January 2009	18134 (g)	0.19	12630; 26036	14.85
		4277 (la)	0.46	1810; 10102	3.48
	February 2010	20562 (g)	0.14	15544; 27200	16.83
		6022 (la)	0.30	3390; 10698	4.90
Autumn	May 2009	7088 (g)	0.24	4458; 11271	5.80
		5586 (la)	0.36	2840; 10986	4.38
	April 2010	7799 (g)	0.33	4147; 14644	6.37
		3352 (la)	0.25	2074; 5419	2.70
Winter	June 2009	16920 (g)	0.52	6518; 43920	13.87
		4440 (la)	0.25	2722; 7243	3.53
	September 2009	14911 (g)	0.21	9858; 22554	12.21
		3139 (la)	0.56	1121; 8795	2.57
	July 2010	3961 (g)	0.21	2592; 6053	3.25
		4718 (la)	0.33	2513; 8855	3.79
Annual		9611 (sm)	0.48	3917; 23583	7.89
Annual average		14995 (g)			12.28
		4415 (la)			3.55

Coefficient of variation (CV), a 95% confidence interval and average density in the prediction area are given. Annual average of abundance and density for *L. guanicoe* and large-livestock is indicated at the bottom of the table.

**Figure 3:**
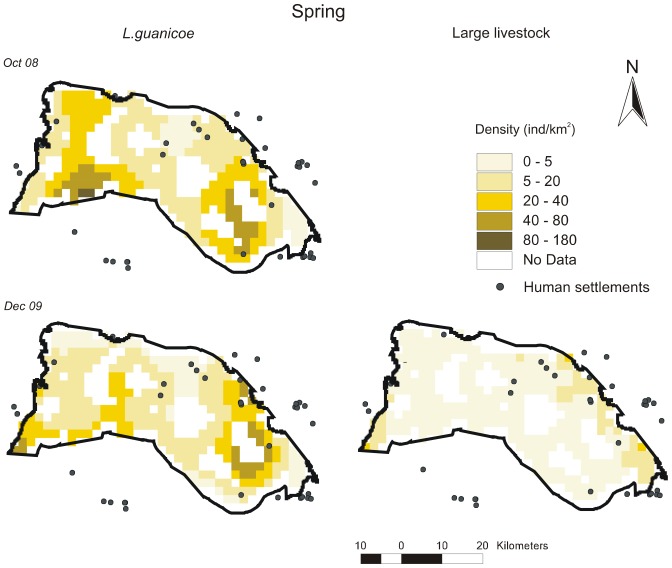
Spatial density distribution maps for *L guanicoe* and large-livestock in spring surveys. Maps for the 1220^2^ prediction area were constructed according to the corresponding best-fit DSMs.

**Figure 4:**
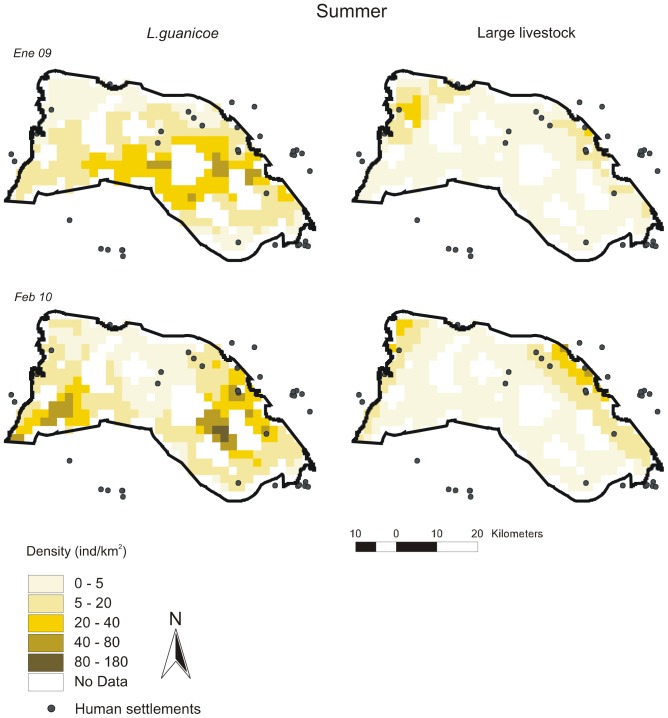
Spatial density distribution maps for *L guanicoe* and large-livestock in summer surveys. Maps for the 1220^2^ prediction area were constructed according to the corresponding best-fit DSMs.

**Figure 5:**
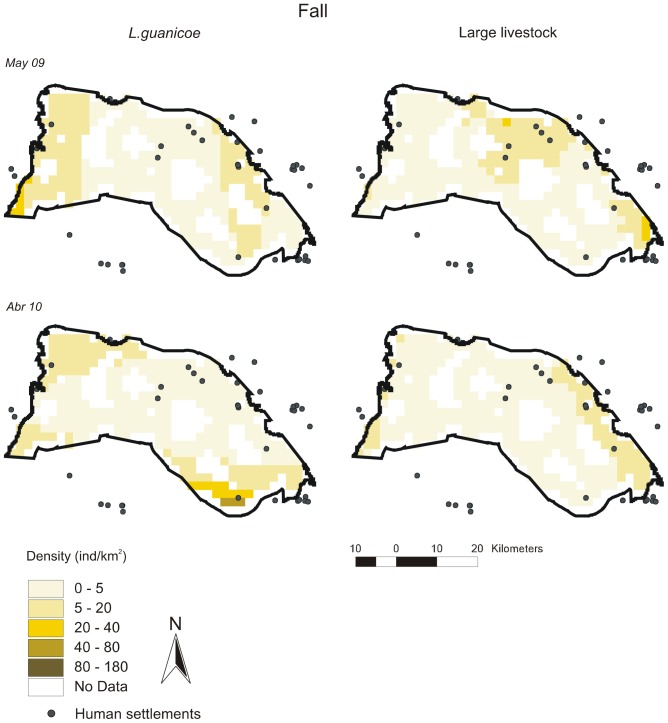
Spatial density distribution maps for *L guanicoe* and large-livestock in fall surveys. Maps for the 1220^2^ prediction area were constructed according to the corresponding best-fit DSMs.

**Figure 6:**
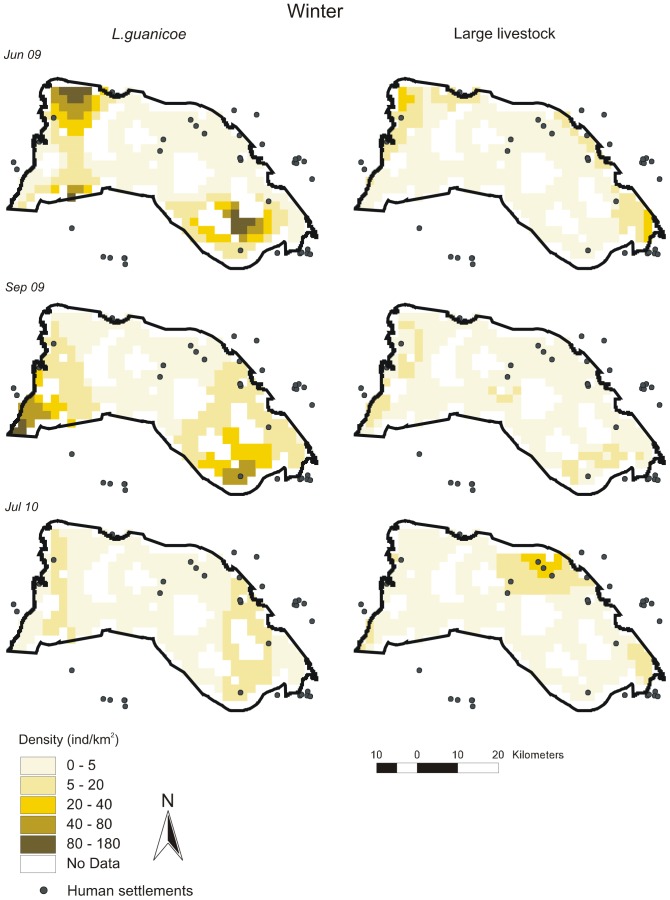
Spatial density distribution maps for *L guanicoe* and large-livestock in winter surveys. Maps for the 1220^2^ prediction area were constructed according to the corresponding best-fit DSMs.

Distribution maps per survey according to best-fit DSMs are given in Fig. 3, 4, 5, 6, 7 for all ungulates. *L.guanicoe* was predicted to be widely distributed in the prediction area both in spring and summer, showing marked high-density zones (between 40 and 180 individuals/km^2^) in the central-southern area (Fig. 3 and 4). In fall, concurrent with low abundance values (Table 3), *L. guanicoe* occurred at low densities towards the periphery of the prediction area (Fig. 5), while in winter animals were in few high-medium density sites (Fig. 6).

**Figure 7:**
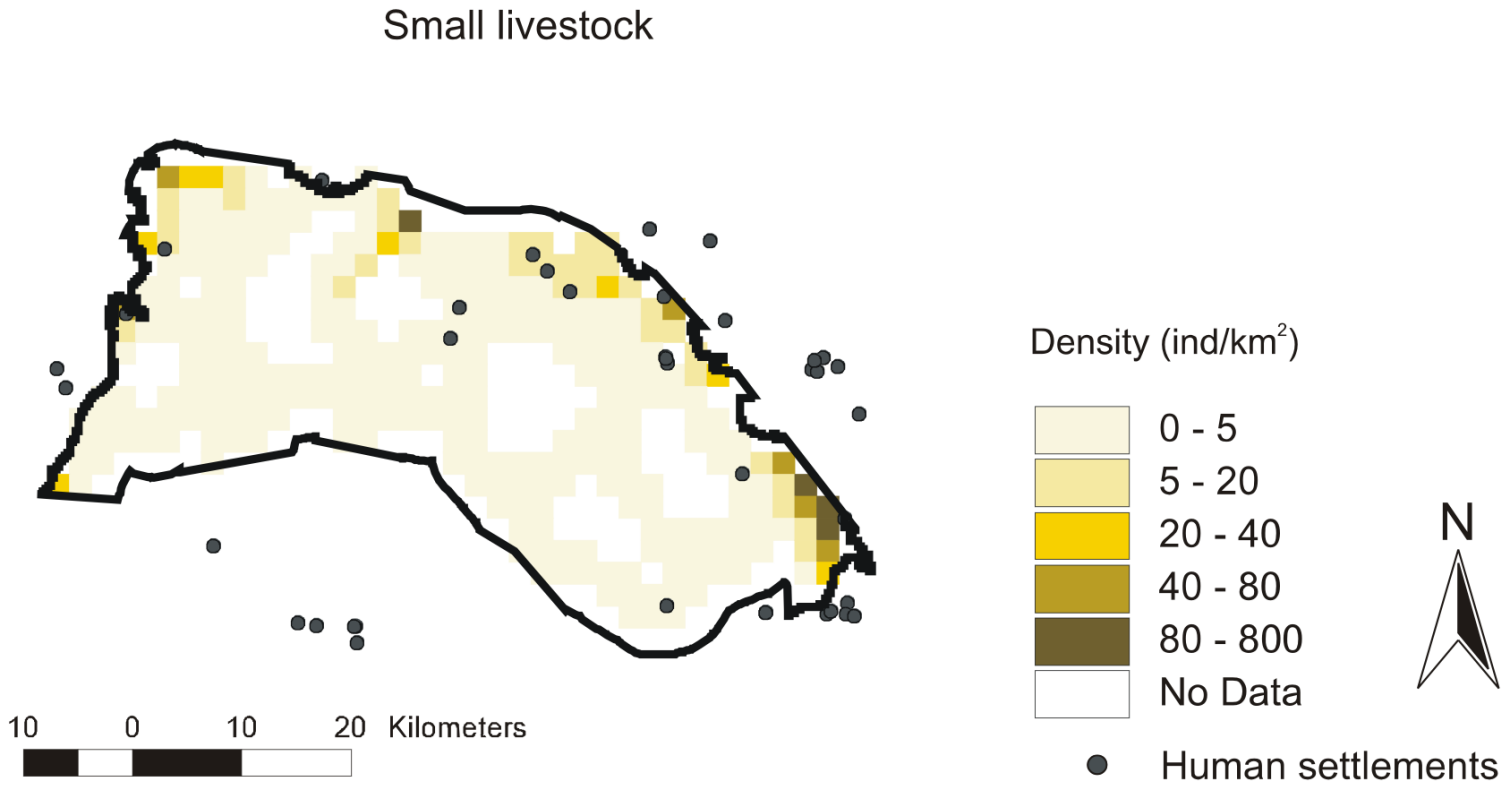
Annual spatial density distribution map for small-livestock. Map for the 1220 km^2^ prediction area were constructed according to the corresponding best-fit DSMs.

Contrary to native species, large-livestock had a more constant spatial distribution, and more similar between seasons. Livestock was restricted to sites close to human settlements in all seasons (large-livestock, Fig. 3 to 6) or annually (small-livestock) (Fig. 7). While large-livestock showed medium values of spatial density (up to 40 individuals/km^2^ for all seasons), goats and sheep occupied few high-density sites (up to 800 individuals/km^2^).

### Co-occurrence patterns

By visually comparing distribution maps of native and domestic herbivores, in all seasons, the highest densities of guanacos (darker colors) were in sites with no or low density of livestock, i.e. the maximum density values of herbivore species did not coincide spatially. All observed covariances in abundance (*U*) between *L.guanicoe* compared to both types of livestock were significantly smaller than expected (*Ū*) from simulated matrices using the IT algorithm (Table 4). Finally, except for July 2010, the SES for the comparison between *L.guanicoe* and small-livestock were always higher than the SES for *L.guanicoe* and large-livestock (Table 4).

**Table 4:** Observed covariance in abundance (*U*) of *Lama guanicoe* compared to large-livestock (g.la) and small-livestock (g.sm) for all surveys and seasons.

Season	Survey	Comparison	*U*	CI.low	CI.up	*Ū*	SES
Spring	Dec-09	g.la	1.07	1.33	1.38	1.35	–26.29
		g.sm	0.95	1.76	1.81	1.79	–58.37
Summer	Jan-09	g.la	0.74	1.40	1.44	1.42	–58.28
		g.sm	0.96	1.80	1.85	1.82	–63.12
	Feb-10	g.la	1.06	1.51	1.54	1.53	–55.90
		g.sm	0.98	1.74	1.79	1.76	–58.27
Fall	May-09	g.la	0.79	1.83	1.88	1.86	–90.77
		g.sm	0.99	1.94	1.97	1.95	–127.06
	Apr-10	g.la	0.93	1.63	1.71	1.67	–35.93
		g.sm	0.99	1.96	1.98	1.98	–177.56
Winter	Jun-09	g.la	1.05	1.46	1.49	1.47	–54.83
		g.sm	0.96	1.84	1.87	1.86	–88.56
	Sep-09	g.la	1.14	1.38	1.42	1.40	–26.77
		g.sm	0.97	1.89	1.93	1.91	–95.86
	Jul-10	g.la	0.80	1.87	1.91	1.89	–104.18
		g.sm	0.99	1.67	1.73	1.70	–51.08

Upper (CI.up) and lower (CI.low) 95% confidence limits using the IT null model algorithm (replicates  =  5000), mean expected *U* value (*Ū*) and the standardized effect size (SES  =  (U–Ū)/sd*_Ū_* ) are given.

## Discussion

Obtaining un-biased, cost-effective estimates of species abundance and distribution was traditionally a difficult task to achieve for large herbivores, and ecologists have continuously searched for more accurate methods. By modeling spatial variation in animal density from standard line-transect data, DSM opens new options for achieving this purpose. Also, by combining DSM with null modeling, our analytical approach provides a new and novel example of how multi-species animal distributions can be characterized and evaluated in relation to potential species interactions. Our methods are scientifically compelling, particularly because they can be used with count data collected at relatively low cost and is also highly useful for researchers worldwide, who conduct counts of animals to simultaneously estimate population sizes, distributions, assess temporal trends and characterize multi-species spatial interactions. Finally, modern model-based analysis methods can improve the planning of conservation strategies, since managers can identify subtle impacts on species, by estimating spatial redistribution of animals as a result of any particular threat [5].

According to our findings, in summer –the breeding season for *L.guanicoe*- sites of predicted high densities coincided with those reported as breeding areas, included in the core area of the reserve [17], [28], [29]. Similarly, high-density sites that were predicted in the south of the study area for June and September 2009 (winter) and April 2010 (fall) indicate the presence of mixed group herds (family groups gathered at the end of the breeding season) moving toward lower-altitude areas of warmer temperatures for the winter [14], [22], [30]. The high concentration of animals predicted for the northwestern portion of the study area in June 2009, which was not observed neither in 2010 nor in previous studies for the same seasons, is consistent with information from telemetry data for the same year (MJ. Bolgeri, unpublished data). These results support the validity of our density model predictions, and make plausible the use of this methodology for terrestrial large mammals, with promising results. Future abundance estimations and model predictive power could further be improved with the inclusion of other spatial co-variables, particularly those concerning resource requirements of the species, in order to better understand the response of the species to the variability of environmental conditions.

We recognize the limitation of having collected data following a particular geographic network (in this case, roads), thus potentially underestimating the number and distribution of any ungulate species that might avoid roads and associated human disturbances. Consequently, we acknowledge that the accuracy of our results is based on the assumption that animal distributions and densities are no different along roads than farther from roads, where counts were not conducted. However, two issues suggest that our results are reliable. First, we did not have a problematic line transect data set [24]. As expected under distance sampling theory, the probability of detecting animals consistently decreased as the distance from the transect increased (Figure S1), suggesting that ungulates did not avoid roads, probably due to the low level of disturbance associated with the roads and tracks in the study area, much of them rarely used during the year. Second, as detailed in methods, we were conservative when predicting abundance and distribution of ungulates outside the surveyed transects, making predictions only in others sites close to roads and within the same elevation interval encompassed by the surveyed transects. If financial issues were not a constraint in sampling design, the analytical approach proposed here, in combination with a stratified sampling scheme and the digital aerial survey counting methodology recently developed [9], would considerably improve the accuracy of results obtained.

The predicted distribution for *L.guanicoe* showed a marked seasonal pattern in both total abundance and spatial distribution. While the total number of guanacos reached its annual maximum in spring and summer, abundance values dropped considerably in fall and winter, decreasing up to 6.5 times. In spring and summer, guanacos occupied a large part of the study area, but concentrating in a few low-medium or medium-high density sites in fall and winter, respectively. Although we cannot identify movements' distances, it seems clear that most of guanaco population moves outside the 1220 km^2^ of the prediction area during the fall-winter period, suggesting a migratory behavior of the species. It has been described that guanaco populations were originally sedentary or migratory -either altitudinal or lateral shift in range- depending on resource supply and snow cover [14]; however, currently it is generally assumed that most of past guanaco migrations have been lost due to the advancement of human pressure. Current guanaco seasonal movements have been described only for Chilean populations, in Torres del Paine National Park [31] and Isla Grande of Tierra del Fuego [32], [33]. Preliminary results of telemetry studies in La Payunia region showed that guanacos move seasonally -between summer and winter ranges- greater distances than those obtained for sedentary populations in Central Patagonia (MJ. Bolgeri, unpublished data, [34]). Our results, in concordance with preliminary telemetry tracking, evidence the maintenance of the seasonal migration process of guanaco populations in La Payunia, an increasingly rare event for terrestrial mammals worldwide [35]. Guanaco migration in this area is probably favored by the absence of physical restrictions such as fences, large towns or other anthropogenic barriers. Migration is an adaptive strategy that allows species to mitigate resource shortage by following seasonally changing food quantities or accessibility, and possibly enables them to escape from predators [36]. The migration of large herbivores has been well documented (see for example [35], [36], [37]), along with the regular seasonal displacements of many bird species [38], but there are also reports of wolves following migratory caribous [39], and rare events of long-distance movements of jackrabbits [40]. Migratory strategies are particularly relevant for population persistence in arid regions, where resources are scarce all year round, with snow covering high elevations several months of the year. Further research must be oriented to identify guanaco migrations routes and movement distances, to evaluate protection degree currently offered by natural reserve area and the presence of potential threats, in order to ultimately develop effective site-specific strategies to maintain this ecological meaningful event [35].

Abundance and spatial distribution patterns of large-livestock, in contrast, varied little throughout the year. The predicted abundance ranged from 3139 to 6022 animals. Both large and small-livestock were mostly distributed in the periphery of the study area near human settlements, as expected by the sedentary management practiced by local people, and also found in other ecosystems (i.e. [41], [42]). Unlike *L.guanicoe*, livestock is highly dependent on water, so their movements are naturally restricted by the possibility of accessing this resource [42], [43], only available in pools set up in human settlements or from the few temporary water courses or water-points present in the area.

The first study of the guanaco population in La Payunia Region 20 years ago [28] estimated a population size of approximately 10000 guanacos for the entire natural protected area, corresponding at that time to 4500 km^2^. Our results suggest that the total abundance of guanacos could be considerably higher at present, with an average annual value close to 15000 individuals, and a maximum value of about 26000, in an area of 1220 km^2^ (approximately one-third of the total Reserve area at that time). Moreover, if we consider that guanacos could be avoiding roads, the estimated population size provided here should be considered a minimum estimate. A later study [18], estimated an average density of 4.89 individuals/km^2^ for an area equivalent to our study area, which was lower than our annual density estimations of 12.28 individuals/km^2^ (Table 4). The differences between our and previous results could be due to two main concurrent causes. Density values in previous studies were estimated from direct animal counts using fixed transects, assuming that all animals within the strip width are recorded. Since it is normally difficult to fulfill this assumption, that methodology may have yielded underestimated density values. The line transect method we used here assumes that the probability of observing animals decreases with the distance from the transect, and consequently, it allows to correct abundance estimates by taking this into account. Thus, the requirement is to record all individuals near the transect line [24]. In addition, this methodology accounts for the group size effect, which as we found (Appendix S2, Table S1), may affect the detection probability and therefore the final density estimates. The methodology based on detection probabilities is therefore considered more robust than fixed transects methods, and is usually preferred for population studies of species as diverse as bivalves [6], fishes [44], small mammals [45], birds [46], foxes [47] and large herbivores [48]. Furthermore, the DSMs used in this study are expected to provide an improvement in the accuracy of abundance estimates compared to conventional design-based models based on distances, since they model part of the spatial variability [3]. The second cause that could account for the differences between our results and those of other authors is a possible population growth of *L. guanicoe* since the creation of the protected area. Puig and collaborators [18] also found that density values in inner sectors of the Reserve were significantly higher in 2002–2003 compared to the values recorded 20 years before (1982–84) for the spring-summer period. The authors conclude that this increase is compatible with an average annual growth of 2%.

According to our prediction, the results of density surface models and distribution maps ensued from them, suggest high spatial segregation between *L. guanicoe* and domestic herbivores. Although *L. guanicoe* used the landscape differently throughout the year, it kept a low overlap with livestock on medium and high-density sites in all seasons, which was confirmed by the co-occurrence analysis. The herbivore distributions in the landscape were non-random, i.e., abundance of *L. guanicoe* and livestock negatively co-varied in all surveys more than expected by chance. Moreover, when we analyzed the co-occurrence of *L. guanicoe* and both types of livestock separately, the degree of segregation of *L. guanicoe* and small-livestock was comparatively stronger than that of *L. guanicoe* and large-livestock in 7 of 8 surveys, as expected. Both results support our hypothesis that less co-occurrence patterns are expected to occur between ecological similar herbivores in order to meet nutritional requirements, probably suggesting a competition mechanism for food-limiting resources. Similar studies of native and domestic herbivores conducted in different ecosystems worldwide agree in that native species change their density and spatial distribution patterns in response to changes in density or movements of livestock (i.e. [2], [49]). In a protected area of the African savanna, for example, sharp seasonal differences were found in the density of wild herbivores between the reserve and livestock farms during a time period of 30 years [2]. Studies conducted in central Patagonia [49] found that guanaco abundance increased by three-fold within 3 years after sheep removal, suggesting that guanacos reacted rapidly to changes in management.

Finally, although competition for food-limiting resources are plausible mechanisms behind our co-occurrence results, another, not necessary alternative, explanation needs to be considered. Species could be associated with different abiotic features of the landscape, which may lead to less co-occurrence than expected by chance. Different environmental features such as slope, elevation, proximity to rivers or shelter, plant productivity and spatial distribution of preferred food, in combination with differential accessibility of species, could also reflect “habitat checkerboards” [50]. A third possibility is that patterns of segregation and aggregation might be due to a combination of both biotic interactions and environmental factors. For example, guanacos may be selecting areas that both avoid livestock and at the same time are preferred habitats. Disentangling which factor or factors contribute most strongly to the observed multi-species animal distributions has to be done by considering a much larger suite of predictor variables that may influence each species' distribution, and consequently comparing values of those predictor variables where animals are distributed with areas where the animals don't occur. A recent study in the same area showed that habitat selection of *L. guanicoe* is explained both by negative interaction with small-livestock, but also in response to vegetation productivity and human pressure, although these conclusions were based on presence-absence data [21]. Except for low density and cryptic species, abundance data is expected to be superior because more information of species is collected at each site, deriving in more ecologically meaningful conclusions, and better ground for management decisions [51]. A mechanistic understanding of whether different species of ungulates are reacting behaviorally to the presence of other species, and adjusting their distributions accordingly, can only be obtained through a manipulative study where domestic ungulates are deliberately moved through the study area, and the distributional response of native species then documented [52], [53]. Finally, other patterns of segregation and aggregation might have been detected with count data collected across the entire study area under a stratified random sampling method that included sampling areas beyond roads. Future research must combine all of this important points to better understand population dynamics of species of conservation interest.

## Supporting Information

Figure S1 **Histograms for distance data grouped into distance intervals.** Solid line represents the best-fit detection function (hazard rate) for *L. guanicoe*, large and small livestock (Table 2).
(TIF)

Table S1 **Summary of the set of models of the detection function for** ***L. guanicoe*** **and domestic herbivores.** The best-fit models are indicated with *. Akaike’s Information Criterion (AIC), its difference with the best-fit model (ΔAIC) and the value of Cramer-von Mises test (*P*) are given.
(DOC)

Table S2 **All density surface models (DSM) tested for herbivores in all surveys.** Degrees of freedom (df), Generalized Cross Validation (GCV) and percentage of explained deviance are given. The best-fit models are indicated with *.
(DOC)

Appendix S1 **Links of newspaper and scientific notes.** Repercussions of Santa Cruz initiative of declaring guanacos as pest species.
(DOC)

Appendix S2 **Adjusting detection function.**
(DOC)
