# Supplementary figures and images for: Spatial and Seasonal Dynamic of Abundance and Distribution of Guanaco and Livestock: Insights from Using Density Surface and Null Models
Source: PLoS One. 2014 Jan 22;9(1):e85960. doi: 10.1371/journal.pone.0085960 (PMC3899089; doi:10.1371/journal.pone.0085960)

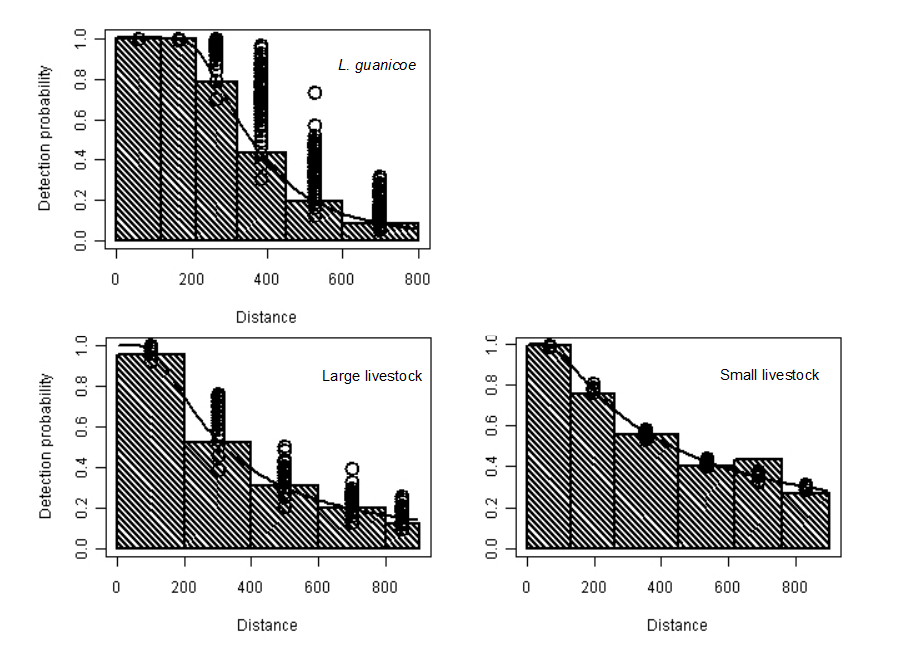

Supplement: Figure S1 — Histograms for distance data grouped into distance intervals. Solid line represents the best-fit detection function (hazard rate) for L. guanicoe, large and small livestock (Table 2). (TIF) [file pone.0085960.s001.tif]
