# Supplementary material for: Spatial and Seasonal Dynamic of Abundance and Distribution of Guanaco and Livestock: Insights from Using Density Surface and Null Models
Source: PLoS One. 2014 Jan 22;9(1):e85960. doi: 10.1371/journal.pone.0085960 (PMC3899089; doi:10.1371/journal.pone.0085960)
Supplement: Table S1 — Summary of the set of models of the detection function for L. guanicoe and domestic herbivores. The best-fit models are indicated with *. Akaike’s Information Criterion (AIC), its difference with the best-fit model (ΔAIC) and the value of Cramer-von Mises test (P) are given. (DOC) [file pone.0085960.s002.doc]

**Table S1**. Summary of the set of models of the detection function for *L. guanicoe* and domestic herbivores. The best-fit models are indicated with *. Akaike’s Information Criterion (AIC), its difference with the best-fit model (ΔAIC) and the value of Cramer-von Mises test (*P*) are given.

| **Species /group of species** | **MODEL** | **AIC** | **ΔAIC** | ***P***  **(Cramer-von Mises test)** |
| --- | --- | --- | --- | --- |
| guanaco | g_1_: half-normal | 27990.36 | 88.4 | 0.05 |
|  | g_2_: hazard rate | 27961.16 | 59.02 | 0.76 |
|  | g_3_: half-normal (group size) | 27912.91 | 10.95 | 0.04 |
|  | g_4_: hazard rate (group size)* | 27901.96 | 0.00 | 0.46 |
| large-livestock | g_5_: half -normal | 9984.48 | 50.33 | <0.001 |
|  | g_6_: hazard rate | 9937.51 | 3.36 | 0.39 |
|  | g_7_: half -normal (group size) | 9973.89 | 39.74 | <0.001 |
|  | g_8_: hazard rate (group size)* | 9934.15 | 0.00 | 0.36 |
| small-livestock | g_9_: half -normal* | 889.,7 | 0.00 | 0.70 |
|  | g_10_: hazard rate * | 891.01 | 1.31 | 0.97 |
|  | g_11_: half -normal (group size) | 896.45 | 6.75 | 0.01 |
|  | g_12_: hazard rate (group size) | 892.99 | 3.29 | 0.97 |
