# Supplementary material for: Spatial and Seasonal Dynamic of Abundance and Distribution of Guanaco and Livestock: Insights from Using Density Surface and Null Models
Source: PLoS One. 2014 Jan 22;9(1):e85960. doi: 10.1371/journal.pone.0085960 (PMC3899089; doi:10.1371/journal.pone.0085960)
Supplement: Table S2 — All density surface models (DSM) tested for herbivores in all surveys. Degrees of freedom (df), Generalized Cross Validation (GCV) and percentage of explained deviance are given. The best-fit models are indicated with *. (DOC) [file pone.0085960.s003.doc]

**Table S2.** All density surface models (DSM) tested for herbivores in all surveys. Degrees of freedom (df), Generalized Cross Validation (GCV) and percentage of explained deviance are given. The best-fit models are indicated with *.

| **Sampling** | | **Model** | **Co-variables** | **df** | **GCV** | **Expl. Dev. (%)** |
| --- | --- | --- | --- | --- | --- | --- |
| *L. guanicoe* | | | | | | |
|  | October2008 (spring) | hg1 | s(*x*) +s(*y*)***** | 6.59 | 120.75 | 28.2 |
|  |  | hg2 | s(*x*) +s(*y*) + s(*d*) | 5.724 | 130.24 | 19.4 |
|  |  | hg3 | s(*d*) | 1 | 124.27 | 5.7 |
|  | January 2009 (summer) | hg4 | s(*x*) +s(*y*) | 3.177 | 54.87 | 34.8 |
|  |  | hg5 | s(*x*) +s(*y*) + s(*d*)***** | 7.034 | 52.41 | 43.7 |
|  |  | hg6 | s(*d*) | 1 | 72.72 | 3.9 |
|  | May 2009 (fall) | hg7 | s(*x*) +s(*y*)***** | 4.656 | 41.07 | 24.3 |
|  |  | hg8 | s(*x*) +s(*y*) + s(*d*) | 3 | 41.64 | 18.8 |
|  |  | hg9 | s(*d*) | 1.131 | 45.62 | 5.4 |
|  | June 2009 (winter) | hg10 | s(*x*) +s(*y*)***** | 7.814 | 66.77 | 50.2 |
|  |  | hg11 | s(*x*) +s(*y*) + s(*d*) | 9.111 | 68.54 | 51.3 |
|  |  | hg12 | s(*d*) | 2.185 | 98.52 | 10.7 |
|  | September 2009 (winter) | hg13 | s(*x*) +s(*y*)***** | 4.719 | 89.19 | 36.5 |
|  |  | hg14 | s(*x*) +s(*y*) + s(*d*) | 5.462 | 91.79 | 36.3 |
|  |  | hg15 | s(*d*) | 1 | 115.35 | 7 |
|  | December 2009 (spring) | hg16 | s(*x*) +s(*y*)***** | 8.839 | 83.60 | 27 |
|  |  | hg17 | s(*x*) +s(*y*) + s(*d*) | 9.734 | 84.35 | 28.8 |
|  |  | hg18 | s(*d*) | 1.768 | 86.24 | 3.6 |
|  | February 2010 (summer) | hg19 | s(*x*) +s(*y*) | 6.726 | 87.03 | 42.8 |
|  |  | hg20 | s(*x*) +s(*y*) + s(*d*)***** | 8.679 | 81.08 | 50.4 |
|  |  | hg21 | s(*d*) | 2.823 | 116.75 | 12.2 |
|  | April 2010 (fall) | hg22 | s(*x*) +s(*y*)***** | 3.149 | 54.76 | 31.4 |
|  |  | hg23 | s(*x*) +s(*y*) + s(*d*) | 4.08 | 56.52 | 31.3 |
|  |  | hg24 | s(*d*) | 1 | 74.01 | 3.1 |
|  | July 2010 (winter) | hg25 | s(*x*) +s(*y*)***** | 4.85 | 46.91 | 18.1 |
|  |  | hg26 | s(*x*) +s(*y*) + s(*d*) | 7.248 | 47.494 | 23.8 |
|  |  | hg27 | s(*d*) | 1.623 | 46.65 | 9.2 |
| *Large-livestock* | | | | | | |
|  | January 2009 (summer) | hm4 | s(*x*) +s(*y*) | 7.182 | 28.25 | 46.9 |
|  |  | hm5 | s(*x*) +s(*y*) + s(*d*)***** | 9.105 | 25.94 | 51.6 |
|  |  | hm6 | s(*d*) | 2.822 | 31.98 | 30.1 |
|  | May 2009 (autumn) | hm7 | s(*x*) +s(*y*) | 7.739 | 12.42 | 62.9 |
|  |  | hm8 | s(*x*) +s(*y*) + s(*d*)***** | 7.775 | 11.65 | 65.3 |
|  |  | hm9 | s(*d*) | 2.653 | 16.42 | 41.5 |
|  | June 2009 (winter) | hm10 | s(*x*) +s(*y*) | 5.307 | 11.66 | 64.1 |
|  |  | hm11 | s(*x*) +s(*y*) + s(*d*)***** | 6.461 | 11.44* | 66.2 |
|  |  | hm12 | s(*d*) | 2.017 | 16.33 | 43.8 |
|  | September 2009 (winter) | hm13 | s(*x*) +s(*y*) | 3.649 | 25.95 | 25.7 |
|  |  | hm14 | s(*x*) +s(*y*) + s(*d*)***** | 4.882 | 20.85* | 42.8 |
|  |  | hm15 | s(*d*) | 2.875 | 22.06 | 35.2 |
|  | December 2009 (spring) | hm16 | s(*x*) +s(*y*) | 4.643 | 27.84 | 36.8 |
|  |  | hm17 | s(*x*) +s(*y*) + s(*d*)***** | 6.611 | 26.76* | 43.3 |
|  |  | hm18 | s(*d*) | 2.07 | 29.95 | 25.9 |
|  | February 2010 (summer) | hm19 | s(*x*) +s(*y*)***** | 4.292 | 22.91* | 47.8 |
|  |  | hm20 | ss(*x*) +s(*y*) + s(*d*) | 8.274 | 23.07 | 54.3 |
|  |  | hm21 | s(*d*) | 1.697 | 27.57 | 31.4 |
|  | April 2010 (autumn) | hm22 | s(*x*) +s(*y*) | 4.333 | 15.08 | 49.4 |
|  |  | hm23 | s(*x*) +s(*y*) + s(*d*)***** | 6.695 | 14.70 | 54.6 |
|  |  | hm24 | s(*d*) | 2.603 | 17.55 | 37.6 |
|  | July 2010 (winter) | hm25 | s(*x*) +s(*y*) | 5.93 | 13.83 | 50.3 |
|  |  | hm26 | s(*x*) +s(*y*) + s(*d*)***** | 9.102 | 13.76 | 56 |
|  |  | hm27 | s(*d*) | 1.893 | 16.52 | 31.9 |
| *Small-livestock* | | | | | | |
|  | Annual | he1 | s(*x*) +s(*y*) | 7.259 | 30.35 | 35.4 |
|  |  | he2 | s(*x*) +s(*y*) + s(*d*)***** | 10.264 | 29.37 | 38.1 |
|  |  | he3 | s(*d*) | 1.731 | 42.05 | 8.64 |
