# Supplementary material for: Spatial and Seasonal Dynamic of Abundance and Distribution of Guanaco and Livestock: Insights from Using Density Surface and Null Models
Source: PLoS One. 2014 Jan 22;9(1):e85960. doi: 10.1371/journal.pone.0085960 (PMC3899089; doi:10.1371/journal.pone.0085960)
Supplement: Appendix S1 — Links of newspaper and scientific notes. Repercussions of Santa Cruz initiative of declaring guanacos as pest species. (DOC) [file pone.0085960.s004.doc]

**Appendix S1.** Links of newspaper and scientific notes about repercussions of Santa Cruz initiative of declaring guanacos as pest species.

<http://www.diariojornada.com.ar/Noticia/Default.aspx?id=52332>, <http://www.lanacion.com.ar/1507622-los-guanacos-una-especie-perjudicial>.

http://www.camelidosgecs.com.ar/pdf/carta_cap_23_08_12.pdf
