# Supplementary material for: Spatial and Seasonal Dynamic of Abundance and Distribution of Guanaco and Livestock: Insights from Using Density Surface and Null Models
Source: PLoS One. 2014 Jan 22;9(1):e85960. doi: 10.1371/journal.pone.0085960 (PMC3899089; doi:10.1371/journal.pone.0085960)
Supplement: Appendix S2 — Adjusting detection function. (DOC) [file pone.0085960.s005.doc]

**Appendix S2. Adjusting detection function**

We estimated *g(y),* from perpendicular distances of each observation. Following Thomas et al. (2010), we considered two functions as candidate detection functions:

1. The half-normal function, with one parameter:
2. $g(y)=\exp\left(\frac{-y^{2}}{2\sigma^{2}}\right)$The hazard-rate function, with two parameters:

where *σ* is a scale parameter, and *b* a shape parameter.

We followed this procedure independently for each species (or species group in the case of livestock) and survey.

Since probability of detecting animals may be high for large groups, we compared models with and without group size as covariable (Marques and Buckland 2004). To do this, we considered the scale parameter *σ* as a constant variable, or as a function of the co-variable “group size” (*s*), expressed as *log(s),* according to the following relation:

We used Akaike’s Information Criteria (Burnham and Anderson 2002) to select the best model, but also considering Cramer-von Mises’ goodness-of-fit test and visually exploring frequency histograms of distances (Buckland et al. 2001). To correct bias from possible evasive movements of animals or rounding in distance records (Buckland et al. 2001) we then re-adjusted the models by grouping the observations into manually defined distance intervals and using a chi-square goodness-of-fit test.

The best model of detection function for all herbivores was the Hazard-rate function, including group size as co-variable for guanaco and large-livestock (Table S1). The absence of a group size effect for small livestock probably indicate that it is easier to detect goats and sheep at large distances (although they are individually small animals) than other major ungulates because they are always in larger visible groups (mean group size = 53.2). For small-livestock, the half-normal (g_9_) and hazard rate (g_10_) models were equally supported by the data (ΔAIC <2). We chose the g_10_ model because it showed better goodness of fit (P=0.97, Table S1).

**Literature cited**

Burnham, K.P. & Anderson, D.R. (2002). *Model selection and multimodel inference: a practical information-theoretic approach*. New York: Springer-Verlag.

Buckland, S.T., Anderson, D.R., Burnham, K.P., Laake, J.L., Borchers, D.L. & Thomas, L. (2001). *Introduction to Distance Sampling*. Oxford: Oxford University Press.

Marques, F.F.C. & Buckland, S.T. (2004). Covariate models for the detection function. In *Advanced Distance Sampling*. Buckland, S.T., Anderson, D.R., Burnham, K.P., Laake, J.L., Borchers, D.L. & Thomas, L. (Eds). Oxford: Oxford University Press.

Thomas, L., Buckland, S.T., Rexstad, E., Laake, J.L., Strindberg, S., Hedley, S.L., Bishop, J.R., Marques, T. & Burnham, K.P. (2010). Distance software: design and analysis of distance sampling surveys for estimating population size. *J. Appl. Ecol.* **47**, 5–14.
